# Atypical response with bone pseudoprogression in a patient receiving nivolumab for advanced cutaneous squamous cell carcinoma

**DOI:** 10.1186/s40425-018-0444-5

**Published:** 2018-11-27

**Authors:** Leandro J. C. Oliveira, Aline B. L. Gongora, Felipe G. Barbosa, Carlos H. dos Anjos, Rodrigo R. Munhoz

**Affiliations:** 1 Oncology Center - Hospital Sírio Libanês, Rua Dona Adma Jafet, 91. 2nd floor. Building A, São Paulo, 01308-050 Brazil; 2 Radiology and Nuclear Medicine Service - Hospital Sírio Libanês, São Paulo, Brazil; 3 Oncology Center - Hospital Sírio Libanês, Brasília, Brazil; 4 Oncology Service, Instituto do Câncer do Estado de São Paulo, Universidade de São Paulo, São Paulo, Brazil

## Abstract

Currently, there is no established standard of care for patients with metastatic CSCC. Based on the mechanisms of CSCC carcinogenesis has been postulated that these tumors may be amenable to PD-1/PD-L1 blockade.

This case illustrates a patient with CSCC with nodal involvement and pulmonary metastases, refractory to two lines of platinum-based regimens and salvage surgery, for whom treatment with nivolumab was recommended. His clinical course was marked by an atypical pattern of response, with initial reduction of soft tissue/visceral lesions, yet development of new bone findings, followed by overall improvement in subsequent scans and sustained disease control upon treatment continuation.

The case of patient with metastatic CSCC, refractory, received immunotherapy and evolved with atypical pattern of response.

## Introduction

Cutaneous SCC (CSCC) is among the most frequent malignancies worldwide and accounts for approximately 20% of all cutaneous neoplasms [1, 2]. Although the incidence of CSCC has increased over the past decades, accurate estimates of the incidence and prevalence of CSCC may be biased by underreporting and misdiagnosis. A systematic review published in 2012 reunited 75 studies from different geographic regions. The highest incidence was observed in Australia (up to 1035 cases/100.000 persons-year), but elevated rates were also reported in the United States (up to 290 cases /100.000 persons-year) and Europe (34.4 cases /100.000 persons-year for males and 15 /100.000 persons- years for females) [3].

Surgical excision remains the treatment of choice for the majority of the patients. Due to a pronounced sensitivity to radiation therapy, this treatment modality may be considered for individuals who are poor surgical candidates, those with locally advanced disease or in situations in which excision followed reconstruction would be cosmetically/functionally unacceptable. Alternatively, radiation therapy with adjuvant intent is often indicated when tumor margins are positive, in the presence of perineural invasion or involvement of adjacent structures or in the setting of recurrent disease [4].

Besides an estimated risk of nodal involvement by CSCC of 3,7 - 5,4%, distant metastases may develop in the lungs, liver, brain, bones and other cutaneous locations, leading to an ominous prognosis [5]. Currently, there is no established standard of care for patients with metastatic CSCC, and treatment decisions are largely based on extrapolations from regimens applicable to non-cutaneous SCC. Several agents have shown some activity in small, prospective trials or retrospective series, including cytotoxic chemotherapy (i.e cisplatin, 5-fluorouracil, bleomycin, methotrexate and doxorubicin), 13-cis-retinoic acid (13cRA) and interferon-2a [6]. In addition to standard chemotherapy, monoclonal antibodies against the *epidermal growth factor receptor* (EGFR) also have demonstrated activity in patients with CSCC. In a prospective study, cetuximab resulted in an overall disease control at 6 weeks of 69% and an objective response rate of 28%. However, benefit is often short-lived. Also, it is important to note that a significant percentage of skin cancer patients are diagnosed at older ages with comorbidities which usually limit to the aapplicability of more intensive treatment regimens [7].

*Programmed cell death receptor 1* (PD-1) is an inhibitory co-receptor expressed by T cells. Binding of PD-1 with its ligands (PD-L1/PD-L2) suppresses anti-tumor responses [8, 9]. The development of monoclonal antibodies targeting the PD-1/PD-L1 axis, or immune checkpoint blockers, has dramatically changed the treatment of different types of malignancies, including squamous-cell carcinoma of the lung cancer and head and neck cancer, renal cell carcinoma [10–17], and a growing number of indications is being exhaustively investigated. Considering that chronic sun exposure, prior radiation, chronic inflammation and skin injury are major risk factors for CSCC, a significant benefit could emerge from strategies exploring immune-checkpoint blockade due to oncogenic mechanisms that involve pronounced DNA damage and an “inflamed” phenotype [8, 18].

We present a case of a patient developing an atypical pattern of response, marked by bone pseudoprogression during therapy with nivolumab for advanced CSCC, followed by sustained response.

## Case report

A 72-year-old man presented with a history of multiple resections of CSCC located in his face, back and upper extremities. Almost 2 years subsequent to the latest excision, he first noted a palpable right axillary mass. A computed tomography (CT) of the chest revealed a 10 cm right axillary nodal conglomerate, additional supraclavicular lymphadenopathies and pulmonary nodules concerning for metastatic disease. These findings were subsequently confirmed by an FDG-PET/CT, and a right axillary mass biopsy was consistent with moderately-differentiated CSCC. First-line therapy with cisplatin 75 mg/m^2^ combined with 5-fluorouracil 1000 mg/m^2^ days 1–4 was recommended, and accompanied by significant gastrointestinal toxicity (diarrhea/mucositis). Re-staging scans revealed a reduction in size of the previously appreciable lesions. A salvage right axillary lymphadenectomy was attempted at an outside institution, revealing involvement by CSCC of 15 out of 16 resected lymph nodes. Early disease progression led to the indication of second-line therapy with carboplatin AUC 5 and paclitaxel 175 mg/m^2^, given every 21 days. Following cycle 6, repeat imaging showed disease progression in the lungs and multiple lymphadenopathies: right axillary, cervical, retropectoral and mediastinal. Of note, no signs of bone involvement were noted, despite degenerative findings and a previously described fracture attributed to osteoporosis.

**Fig. 1:**
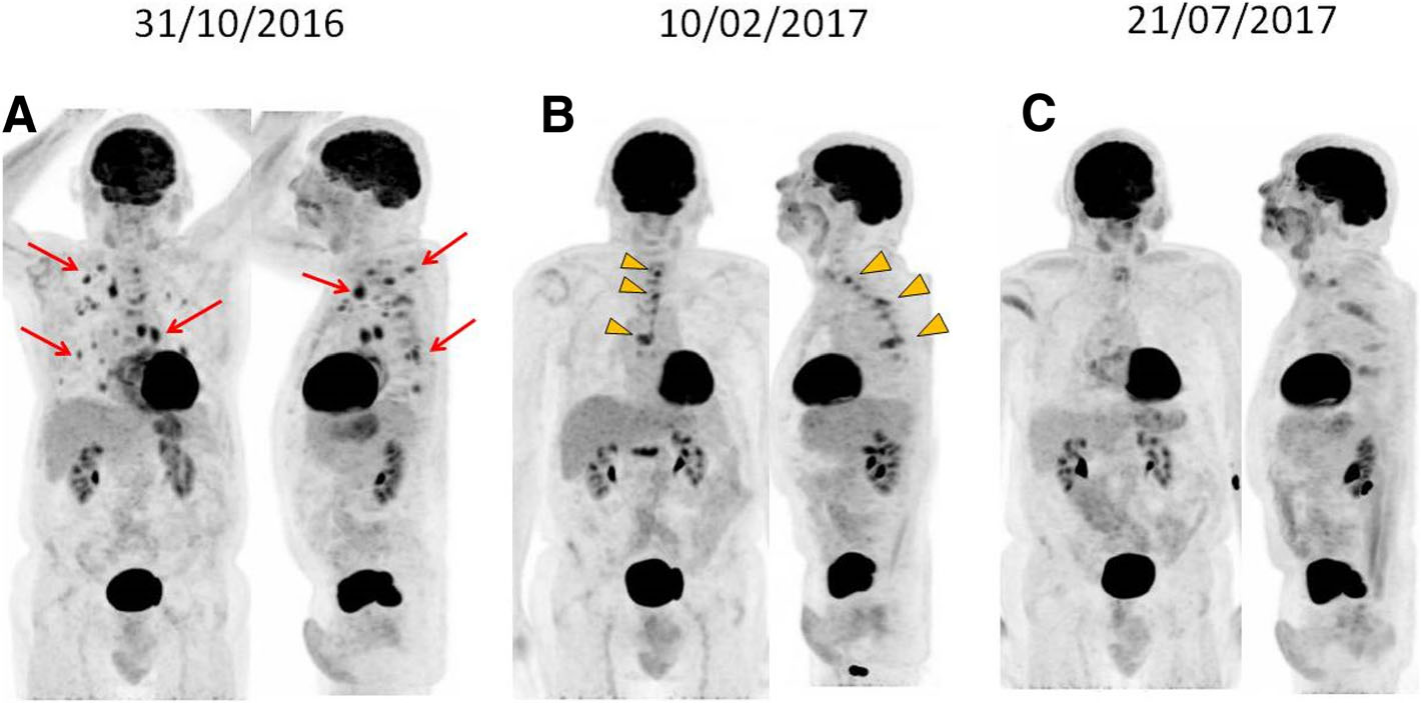
Evolution of pulmonary, mediastinal and vertebral lesions seen in ^18^F-FDG PET/CT’s MIP images during treatment with anti-PD1**.** MIP images (front and lateral view) from different ^18^F-FDG PET/CT’s. **a** Multiple FDG avid lesions in the lungs, mediastinum and thoracic vertebral bodies. **b** Metabolic resolution of the metastatic lung and mediastinal lesions, however there is increased FDG uptake in previous thoracic bone lesions associated with new hypermetabolic bone lesion (arrowhead). **c** Metabolic resolution of most thoracic bone lesions, consistent with partial metabolic response (PMR)

**Fig. 2:**
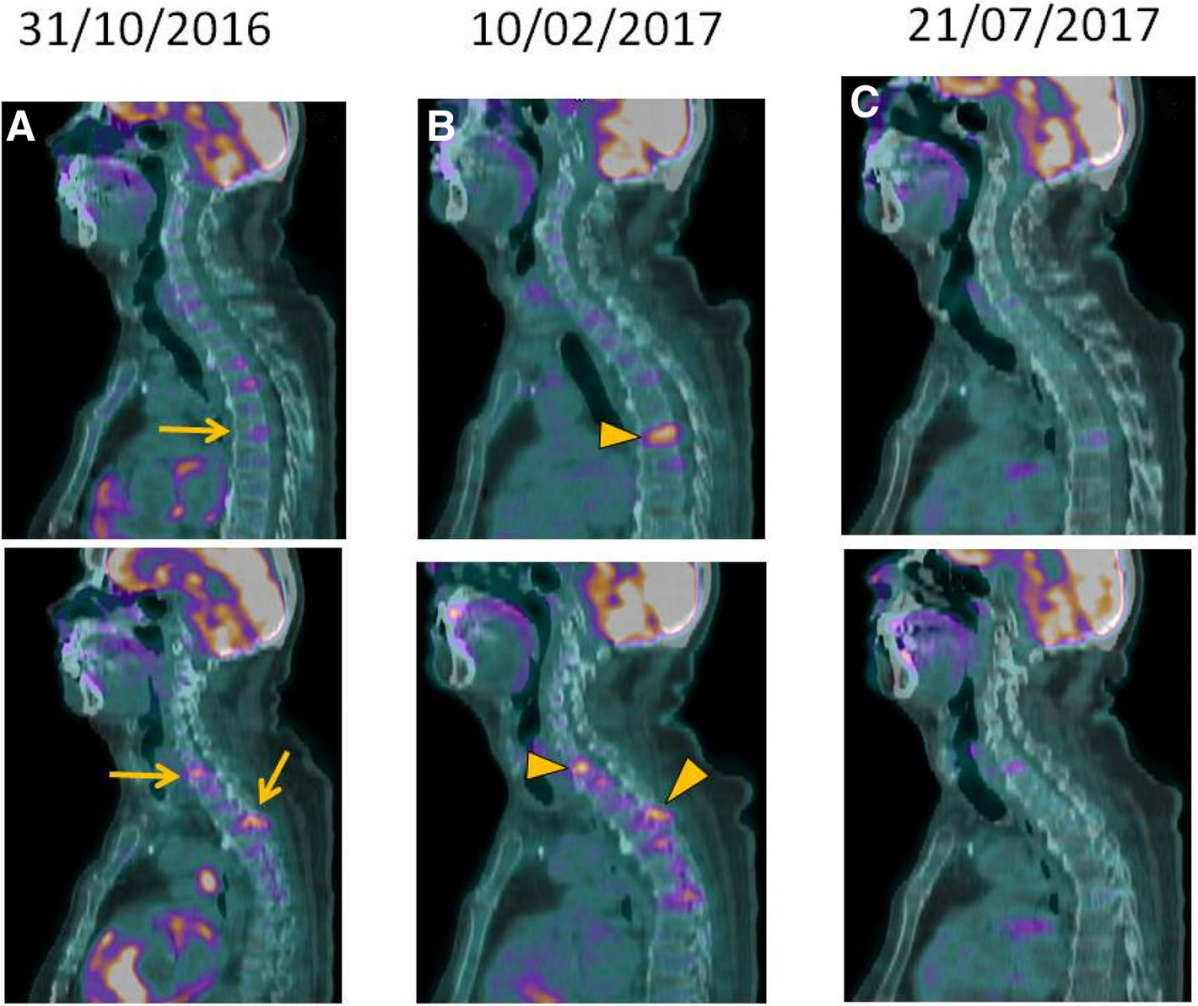
Bone Pseudoprogression after anti-PD1 therapy seen in ^18^F-FDG PET/CT’s. Sagital fused images from different ^18^F-FDG PET/CT’s. **a** Multiple focal FDG uptake in different thoracic vertebrae (T1, T3 and T7) without tomographic focal lesion (arrows), corresponding to bone metastasis. **b** Same corresponding thoracic bone lesions with significant increased uptake (arrowsheads), consistent with metabolic progression disease. **c** Metabolic resolution of most thoracic bone lesions, consistent with partial metabolic response (PMR)

At that point, the patient transitioned his care to our service. Following extensive debate at a multidisciplinary tumor board, decision was made to proceed with third-line nivolumab. After a thorough discussion and clarification regarding the off-label use of the anti-PD-1 agent, the patient received the first dose of nivolumab 3 mg/kg given intravenously every 14 days. Except for non-limiting fatigue, the patient had a remarkable tolerance to nivolumab, accompanied by early clinical response (reduction of right axillary/retropectoral mass). Re-staging PET-CT performed after 3 months of therapy revealed reduction in size and resolution of the metabolic activity of right axillary, cervical, retropectoral and mediastinal lymphadenopathies; pulmonary nodules were no longer appreciable. However, new areas of hypermetabolism were noted in C7, T1, T6 and L1 vertebral bodies, along with new lytic lesions, as well as increased metabolic activity in T3, T4 and T7 pedicles (Figs 1 and 2).

Due to the limited treatment options in this setting and remarkable response of visceral/nodal lesions, and based on a shared decision, continued treatment with nivolumab followed by short-interval repeat (6–8 weeks) scans was recommended. The patient decided to proceed with his treatment locally, at an outside facility, and returned for a reevaluation almost 5 months later, still receiving nivolumab and with no new adverse events. A new PET-CT showed metabolic response in the previously identified bone lesions, now described as sclerotic, and sustained control of pulmonary nodules and lymphadenopathies (Figs 1 and 2). Following more than 12 of nivolumab therapy, the patient continues to endure clinical benefit, with prolonged disease control and good tolerance.

## Discussion

This case illustrates a patient with CSCC with multifocal nodal involvement and pulmonary metastases, refractory to two lines of platinum-based combination regimens and salvage surgery, for whom treatment with nivolumab was recommended. His clinical course was marked by an atypical pattern of response, with initial reduction of soft tissue/visceral lesions, yet development of new bone findings, followed by overall improvement in subsequent scans and sustained disease control upon treatment continuation. In addition to the remarkable radiologic findings, this case highlights the potential for the use of immune-checkpoint blockade for patients with advanced CSCC.

In Brazil, where the patient was treated, non-melanoma skin cancer is among the most frequently diagnosed neoplasms, corresponding to 30% of all malignant tumors in this country [19]**.** There is an unmet need for evidence to guide treatment decisions in patients with advanced CSCC.

Based on the mechanisms of CSCC carcinogenesis and potential markers of immunogenicity [8], it has been postulated that these tumors may be amenable to PD-1/PD-L1 blockade. In a phase 1 study of REGN2810/Cemiplimab, an anti-PD-1 antibody, good tolerability and pronounced activity was documented in 26 patients with metastatic or locally advanced CSCC, with overall response and disease control rates of 46.2 and 69.2%, respectively [18]. Of note, 81% of pre-treatment tumor samples expressed PD-L1 [18]. In a retrospective cases series of patients with CSCC treated with anti-PD-1 agents, five of six patients (83%) achieved a clinical response, with 1 complete and 4 partial responses. The median duration of PD-1 inhibitor exposure was 8 months, and the longest PFS interval was 21 months [20]. More recently, the combined analysis of data from the expansion cohort of a phase I trial (*N* = 26) and from a phase II study (*N* = 59), totaling 85 CSCC patients treated with cemiplimab, suggested objective response rates of 50% in the expansion cohort and 47% in the phase II cohort, with responses exceeding 6 months in 57% of the responders. Of note, treatment was well tolerated, and only 7% of the patients discontinued the treatment due to adverse events [21].

This case also illustrates one the challenges involved in the efficacy assessment is patients treated with immunotherapy. Responses to immune-checkpoint blockade may be considerably diverse from those observed with either cytotoxic chemotherapy or targeted-therapy. Due to mechanisms not fully characterized, an initial growth in tumor size or appearance of new lesions (pseudoprogression) or late tumor regression may occur, followed by delayed, and occasionally durable responses [22–24]. The rate of these so called atypical patterns of response may vary according to the type of monoclonal antibody, disease and response evaluation criteria applied; a recently published systematic review reported an incidence of atypical responses of approximately 6% in patients with distinct solid tumors treated with anti-PD-1 agents [23]. Pseudoprogression in spinal metastases has been described in other cancer types treated with immunotherapy, including a case report of a patient with melanoma treated with immune checkpoint blockade and radiation therapy [25]. Due to such atypical patterns of response, innovative response assessment criteria have been suggested, but not yet fully implemented, including the immune-related response criteria (irRC) and the modified Response Evaluation Criteria in Solid Tumors (iRECIST) [21, 26].

To the best of our knowledge, this is the first report of this pattern of pseudoprogression (new bone lesions) in a patient receiving nivolumab for CSCC and reinforces the need for individualized response criteria and a judicious assessment in order to avoid premature discontinuation of treatment with potential benefits. It also highlights the promising activity demonstrated by immune-checkpoint blockade in patients with CSCC: although prospective, controlled trials are warranted, the use of immunotherapy-based approaches may become the standard of care in the near future for patients affected by this challenging disease.
